# Green Turtle Feeding on Terrestrial Leaves Reveals Energy Pathway From Land to Sea

**DOI:** 10.1002/ece3.70524

**Published:** 2024-11-11

**Authors:** Nathan J. Robinson, Ryan P. Killackey, Veronica Valverde‐Cantillo, Pilar Santidrián Tomillo

**Affiliations:** ^1^ Department of Renewable Marine Resources Institut de Ciències del Mar, Spanish National Research Council—Consejo Superior de Investigaciones Científicas Barcelona Spain; ^2^ Fundación Oceanogràfic de la Comunitat Valenciana, Ciudad de las Artes y las Ciencias Valencia Spain; ^3^ Pollywog Productions LLC Brooklyn New York USA; ^4^ Goldring‐Gund Marine Biology Station The Leatherback Trust Playa Grande Costa Rica; ^5^ Escuela de Biología Universidad de Costa Rica San José Costa Rica; ^6^ Equipo Tora Carey El Jobo Costa Rica; ^7^ Centre Oceanogràfic de les Balears Instituto Español de Oceanografía (IEO, CSIC) Palma de Mallorca Spain

**Keywords:** Costa Rica, diet, drone, sea turtle, trophic, unoccupied aerial vehicle

## Abstract

We report on an adult male green turtle (*Chelonia mydas*) feeding on fallen leaves from a terrestrial tree, frangipani (*Plumeria rubra*), in the waters in front of Cabuyal—a known sea turtle nesting beach—on the north Pacific coast of Costa Rica. This observation, in conjunction with similar reports worldwide, corroborates that terrestrial leaves may be a common food item for green turtles in areas near mangrove forests or coastal deciduous trees. Our observation also indicates that male turtles may feed during reproductive periods if food is available.

## Graphical abstract

We report on an adult male green turtle (*Chelonia mydas*) feeding on fallen leaves from a terrestrial tree, frangipani (*Plumeria rubra*), in the waters of Cabuyal in northern Costa Rica. This observation suggests that male turtles may feed during reproductive periods and illustrates a novel pathway for the transfer of energy from land to sea. As this is the second published report of green turtles feeding on fallen leaves from terrestrial plants, it is possible that this is an opportunistic but widespread phenomenon in areas with coastal deciduous trees.

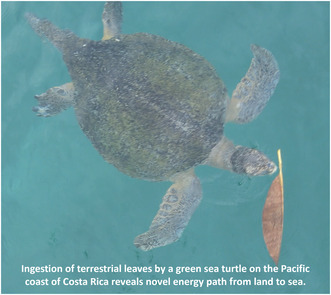

The diet of green turtles (*Chelonia mydas*) has been studied across their circumglobal range (see Esteban et al. 2020 and references within) revealing that green turtles feed predominantly on seagrasses when available but have more diverse diets in cooler regions where seagrasses are not as abundant (Santos et al. 2015). Such plastic foraging behavior may also be expected when turtles are outside of typical foraging habitats, such as when turtles are close to nesting beaches during reproductive periods. A novel food item that has been reported in the diet of green turtles in coastal habitat is the leaves, roots, fruits, and seeds of mangroves and terrestrial plants (e.g., Amorocho and Reina 2007; Nagaoka et al. 2012). Here, we build on these reports with a direct observation of a male East Pacific green turtle feeding on fallen leaves from a terrestrial tree in the waters in front of Cabuyal—a known green turtle nesting beach (Santidrián Tomillo et al. 2015)—on the north Pacific coast of Costa Rica.

On the November 29, 2023, at 07:00, we conducted an opportunistic survey of Cabuyal bay using a DJI Mavic Air 2 (DJI, China). Flying at an altitude of 20 m, we spotted an adult green turtle approximately 200 m from the shoreline. The turtle was identified as a male due to the length of its tail. After filming the animal on the seafloor for a few minutes, the turtle swam toward and ate a large fallen leaf (approximately 25 cm) from a frangipani tree (*Plumeria rubra*) that was floating on the surface of the water (Figure 1A). We continued filming this turtle as it ate five other leaves, approximately one each minute (Figure 1B,C). The turtle only ate the larger frangipani leaves but ignored the smaller red mangrove leaves ( *Rhizophora mangle* ) that were also present. Eventually, the turtle was approached by another male green turtle and after a brief interaction, both turtles left the area. We identified the species of each leaf visually from the video, and our conclusions were supported by the common presence of frangipani and the red mangrove along Cabuyal. The full feeding event can be viewed via Video 1.

**FIGURE 1:**
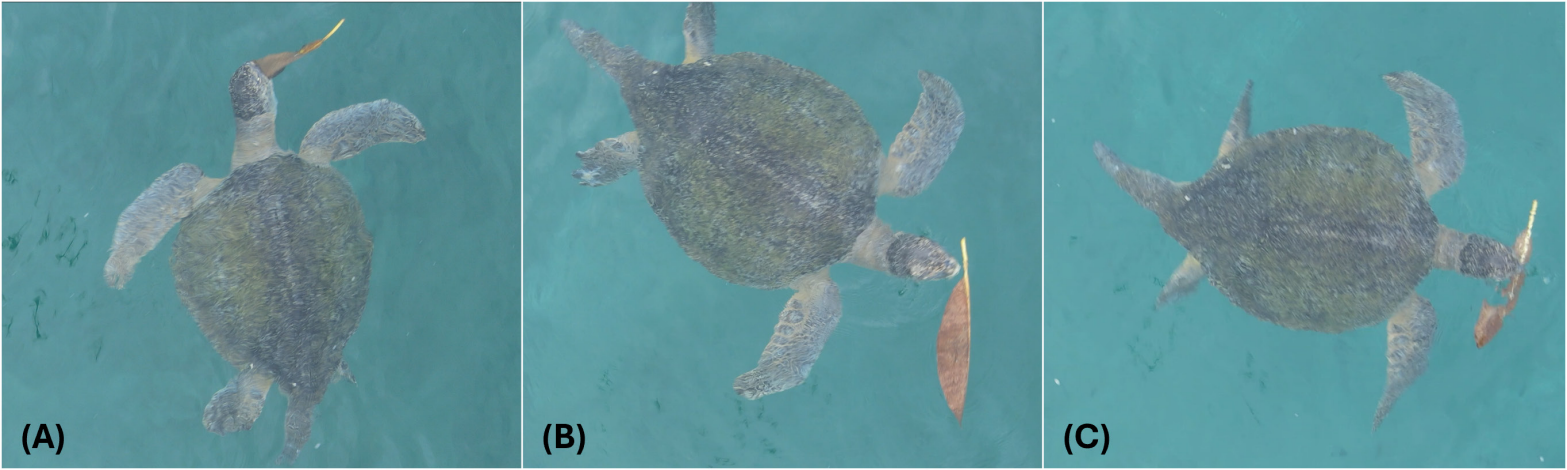
Screenshots of a male green turtle feeding on leaves from the frangipani ( *Plumeria rubra* ) as filmed by a UAV. Each image (A–C) represents a unique event on a different leaf. In total, we observed this individual consuming five different leaves.

**VIDEO 1:** A male green turtle feeding on leaves from the frangipani ( *Plumeria rubra* ) as filmed by a UAV. Video: Video content can be viewed at https://onlinelibrary.wiley.com/doi/10.1002/ece3.70524

This observation, albeit brief, has two important implications. Firstly, it suggests that male turtles, like female turtles (e.g., Myers and Hays 2006; Fuller et al. 2009), may feed during reproductive periods when food is available. This could offset some of the energetic costs associated with reproduction as studies have noted large declines in the body condition, shown via a loss of both adipose and muscle tissue, in male turtles during reproductive periods (Jessop, Hamann, and Limpus 2004). Yet even though this video was recorded during the sea turtle nesting season on Cabuyal, mating pairs had been spotted from the beach in the waters during the preceding weeks, and the turtle appeared to exhibit the typical long tail of a sexually mature male (Casale et al. 2005), we cannot unequivocally confirm that this animal was reproductively active. Indeed, some female green turtles are known to remain residential in the waters of Cabuyal bay even outside of the nesting season (Clyde‐Brockway 2014) and so it is likely that some male turtles also remain in these waters year‐round. Secondly, this observation adds to a growing number of studies reporting that green turtles will occasionally feed on terrestrial plants or mangroves. Such reports span across the west, central, and east Pacific (López‐Mendilaharsu et al. 2005; Arthur and Balazs 2008; Carrión‐Cortez, Zárate, and Seminoff 2010; Limpus and Limpus 2000; Sampson et al. 2018; McDermid et al. 2018) as well as the west Atlantic (Nagaoka et al. 2012; González Carman et al. 2014). As such, this could represent a key mechanism by which terrestrial‐derived nutrients are transferred in marine ecosystems. Indeed, McDermid et al. (2018) demonstrate that in some instances terrestrial leaves could have higher calorie content than more typical food items for green turtles such as seagrass and marine algae even after accounting for the higher quantities of indigestible lignin in terrestrial plants. Not all terrestrial plants may be equally nutritious though and this green turtle appeared to feed selectively on frangipani while ignored the leaves of the red mangrove ( *Rhizophora mangle* ). As frangipani leaves are typically far larger and with substantial stems than those of the red mangrove, frangipani leaves could also be higher in calories and reflect a more optimal feeding choice.

This observation represents a single event from a “one‐off” opportunistic UAV survey on Cabuyal, and so we are unable to comment on the frequency of this behavior at this location. Yet considering that frangipani is a common and deciduous species on coastal beaches throughout Central America, Africa, and Asia and that terrestrially derived plant matter has been observed in the diets of green turtles across the Pacific and Atlantic, we propose that fallen leaves could facilitate an underestimated energy pathway from terrestrial to marine systems that is of key relevance to green turtles.

## Author Contributions

**Nathan J. Robinson:** conceptualization (equal), data curation (equal), formal analysis (equal), investigation (equal), writing – original draft (equal). **Ryan P. Killackey:** methodology (equal), writing – review and editing (equal). **Veronica Valverde‐Cantillo:** project administration (equal). **Pilar Santidrián Tomillo:** supervision (equal), writing – review and editing (equal).

## Conflicts of Interest

The authors declare no conflicts of interest.

## Data Availability

All data are provided in the embedded video attached to this manuscript.
